# TW-SIR: time-window based SIR for COVID-19 forecasts

**DOI:** 10.1038/s41598-020-80007-8

**Published:** 2020-12-31

**Authors:** Zhifang Liao, Peng Lan, Zhining Liao, Yan Zhang, Shengzong Liu

**Affiliations:** 1 School of Computer Science and Engineering, Central South University, Changsha, 410075 China; 2 Nuffield Health Research Group, Nuffield Health, Ashley Avenue, Epsom, Surrey, KT18 5AL UK; 3 Department of Computing, School of Computing, Engineering and Built Environment, Glasgow Caledonian University, Glasgow, G4 OBA UK; 4 Department of Information Management, Hunan University of Finance and Economics, Changsha, 410075 China

**Keywords:** Computational biology and bioinformatics, Computational models

## Abstract

Since the outbreak of COVID-19, many COVID-19 research studies have proposed different models for predicting the trend of COVID-19. Among them, the prediction model based on mathematical epidemiology (SIR) is the most widely used, but most of these models are adapted in special situations based on various assumptions. In this study, a general adapted time-window based SIR prediction model is proposed, which is characterized by introducing a time window mechanism for dynamic data analysis and using machine learning method predicts the basic reproduction number and the exponential growth rate of the epidemic. We analyzed COVID-19 data from February to July 2020 in seven countries–––China, South Korea, Italy, Spain, Brazil, Germany and France, and the numerical results showed that the framework can effectively measure the real-time changes of the parameters during the epidemic, and error rate of predicting the number of COVID-19 infections in a single day is within 5%.

## Introduction

Since the outbreak of COVID-19, the epidemic has spread rapidly in many countries and regions in the world, the World Health Organization declared COVID-19 as a Public Health Emergency of International Concern (PHEIC) on January 30, 2020. According to data released by Johns Hopkins University, there are 37,213,592 confirmed cases and 1,072,959 deaths in 188 countries and regions around the world on October 11, 2020. In order to reduce the impact of COVID-19, forecasting trend of COVID-19, such as COVID-19 peak and stage of its spread, is of great significance for the government to formulate prevention and control strategies, take timely measures, and allocate medical resources. There have been many studies to predict the development trend of the epidemic in various countries and regions. These studies can be roughly divided into three categories: statistical modeling methods, AI-based methods and mathematical epidemic models.

Statistical modeling methods estimate main epidemic parameters through case reports and other data statistics, including the basic reproduction number (${R}_{0}$), the incubation period, serial interval and generation time etc., then use mathematical models such as exponential growth to predict the epidemic curve. Zhao et al.^1^ used an exponential growth model to fit the COVID-19 epidemic curve in China and the results showed that the virus may cause outbreaks. Sanche et al.^2^ collected extensive case reports to estimate key epidemiological parameters, which indicated the need for early and robust control measures to stop the spread of the virus. Pike and Saini^3^ suggest that there may be an effective threshold for public health interventions by calculating future trends in other countries based on mortality statistics observed in China. Li et al.^4^ analyzed the spread of COVID-19 in Hubei Province of China using Gaussian distribution, and predicted the epidemic trends in South Korea, Italy and Iran. The results showed the evolution of the epidemic, and found that the implementation of control would have a significant impact. Tang et al.^5^ estimated the number of basic infections per day in China using time-dependent exposure and diagnosis rates and found that the best measure was sustained and strict self-isolation. There are also some studies using statistical modeling methods^6–10^. However, statistical modeling methods are suitable for roughly estimating the epidemic in the early stage of the epidemic. With the development of the epidemic, these epidemic parameters are constantly changing in different countries and regions, which leads to the kind of prediction is only does not reflect the actual situation of epidemic.

The AI-based prediction methods are emerging methods for predicting COVID-19, which are used to predict how COVID-19 propagates over time and space. Hu et al.^11^ used a modified stacked Auto-Encoder for modelling the transmission dynamics of the epidemics to real-time forecasting the confirmed cases of COVID-19 in China. Yang et al.^12^ divided into the data of SARS outbreak in 2003 with three days as input, and used the long and short-term memory network model (LSTM) for training to predict the new coronavirus outbreak in China mainland. Friston et al.^13^ developed a dynamic causal model of COVID-19 based population dynamics, and this model leveraged Bayesian model comparison. Ardabili et al.^14^ compared and analyzed machine learning and soft computing models for predicting COVID-19 epidemic, and the results showed that the multi-layered perceptron and adaptive network-based fuzzy inference system had high generalization ability for long-term prediction. Arora et al.^15^ used a variety of deep learning models based on LSTM to predict the number of COVID-19 positive reported cases in India, and the results showed that the prediction effect of Bi-LSTM was the best, while the convolutional LSTM was the worst. Although the accuracy of AI-based method is very high and the prediction curve can be fitted well, there are still two problems with AI-based methods. The first one is that the prediction method cannot be trained well to achieve the desired effect because of lacking of the training data, special at the beginning of pandemic^16–18^. The other one is the problem of overfitting in this kind of methods then it may therefore fail to predict reliably. Hence, established mathematical epidemiological models were used to track and forecast in most studies so far.

There are two kind of typical mathematical epidemiological models including SIR and SEIR (Susceptible, Exposed, Infected, and Removed). A number of studies have adapted these two mathematical epidemiological models to meet specific needs and to analyze the transmission dynamics of COVID-19. The modifications of the model are divided into several types: adding a new state or modifying the model parameters on the basis of the original model, integrating additional external data into the model, adding the effects of non-pharmaceutical interventions on the model, etc. Liu et al.^19^ added infected individuals who did not report symptoms based on the SIR model, and used the case data reported in China early to predict the cumulative number of reported cases. The main feature of the model is to model the timing of the government’s main public policies. Peng et al.^20^ and others proposed a generalized SEIR model, re-formulated a new isolation state and considered the effects of preventive measures, and analyzed the epidemic situation in five different regions of China. However, due to the limitations of detection methods and diagnostic criteria, unreported cases and exposed cases are difficult to be estimated and accurate numbers of these cases are difficult to be obtained. These number are regarded as hidden variables in the research process. Sun et al.^21^ developed a time-varying coefficient vSIR model to reflect the changes of model parameters due to the government intervention. Chen et al.^22^ developed a time-dependent SIR model for COVID-19 with undetectable infected persons and used the two finite impulse response filters to track and predict the numbers of infected persons and recovered people in China. As far as the results are concerned, the prediction error is very small, but the training of the model is based on the fact that the data is sufficient, and it is not suitable for the early stage of prediction of the epidemic. Fanelli et al.^23^ used the SIRD model with the death status to predict the epidemic trends in China, Italy and France, and found that the time evolution of COVID-19 has a certain degree of universality and has little connection with geographical changes, but this research study is only based on a simple quantitative model to evaluate the effect of strict epidemic prevention. In addition, other studies have modified the SEIR model, such as considering the population migration data^12^, analyzing the proportion of infected passengers on evacuation flights^24^, and so on. Biswas et al.^25^ used SIR model to fit Chinese data on Euclid network, and the results showed that adding other factors to the model would make the model more complicated. Although these methods of modifying epidemiological models can be used to assess the spread of epidemics and the impact of government intervention strategies, these models require the introduction of additional parameters and depend on many assumptions. At the same time, studies have shown that the increase in the number of unknown parameters in a complex model needs to be estimated by model fitting, which will lead to higher uncertainty in model predictions. Therefore, simple models may be more reliable than complex models in the process of model selection^26^. In addition, considering that it is difficult to ensure the accuracy of the exposed cases data, so we chose the commonly used SIR model in this paper.

In the traditional SIR model, there are two key parameters that reflect the characteristics of the epidemic: infection rate $\upbeta$ and recovery rate $\upgamma$. The infection rate $\upbeta$ indicates that each susceptible population randomly infects $\upbeta$ people per day; the recovery rate $\upgamma$ indicates that the infected person recovers or dies with the probability of $\upgamma$. These two parameters are constant in the traditional SIR model. When applied it to the real world, they are often not able to measure and predict the trend of epidemics. Therefore, many studies have regarded them as functions that change over time^21,22^.

However, due to the different epidemic prevention and control measures in different countries and regions and the development of the epidemic, the manually selected functions are not applicable to the real-time changes of parameters. To reflect this change in key parameters of the infectious disease model, we propose to use a time window to dynamically measure key parameters and on a daily basis, taking into account the different levels of development resulting from the containment measures taken in different countries and regions during the course of the outbreak. The time window refers to the previous period of the day, so that the measurement model parameters can be adapted to different countries and regions. While the $\upbeta$ and $\upgamma$ cannot fully measure the virus in the process of transmission capacity, usually we will use the basic number ${R}_{0}$ to reflect the evolution of the epidemic situation. At the same time, we also used the exponential growth rate as an indicator to reflect the exponential growth during virus transmission. By combining these two indicators, we can track and predict $\upbeta$ and $\upgamma$. Based on this idea, we propose a time-window SIR prediction model (TW-SIR), which can capture and track and predict the dynamic changes of epidemic parameters in real time. We applied TW-SIR model on the COVID-19 historical data in China, South Korea, Italy, Spain, Brazil, Germany and France, and we are interested in addressing the following three important questions for COVID-19.

- *RQ1: How does the TW-SIR model perform in measuring the *${R}_{0}$* and exponential growth rate in the process of epidemic? Compared with the formula derivation method, is the parameter measurement of TW-SIR more reasonable and effective?*
- *RQ2: How effective is the prediction of the TW-SIR model in epidemic COVID-19?*
- *RQ3: Can TW-SIR adapt to the second wave of infection?*

The results of our numerical results analysis are encouraging. The results show that the model can effectively measure the real-time changes of parameters during the spread of epidemics, including the basic number of infections ${R}_{0}\left(t\right)$ and exponential growth rate $Ex(t)$. Our experiments demonstrate that TW-SIR perform better than the formula derivation method in the parameter measurement. And the error rate of predicting the number of COVID-19 infections in a single day is within 5%. At the same time, the model can adapt to the second wave of infection which traditional SIR model cannot do. This study is of great significance for understanding the spread of COVID-19 and guiding the designation of control strategies and measures.

The rest of this paper is organized as follows: in the second section, we propose the TW-SIR model. In the third section, we conducted some numerical experiments and analyzed the experimental results to illustrate the effectiveness of our model. Then, in “Discussion”, we made some discussions and suggestions. Finally, the last section is a summary of the paper.

## Methods

### SIR epidemic model

The susceptibility-infection-recovery (SIR) model^27^ is one of the simplest and commonly used epidemic models. The model consists of three compartments: $S$: the number of susceptible individuals, $I$: the number of infectious individuals, $R$ for the number of removed (and immune) or deceased individuals. The SIR epidemic model can be expressed by following set of ordinary differential equations (ODE):

1 $$\frac{dS(t)}{dt}=-\frac{\beta I(t)S(t)}{N}$$

2 $$\frac{dI(t)}{dt}=\frac{\beta I(t)S(t)}{N}-\gamma I(t)$$

3 $$\frac{dR(t)}{dt}=\gamma I(t)$$

4 $$N=S\left(t\right)+I\left(t\right)+R(t)$$

Among them, $S\left(t\right)$, $I\left(t\right)$ and $R\left(t\right)$, respectively represent the functions of $S$, $I$ and $R$ related to time $t$, and their sum satisfies Eq. (4); $N$ represents the total number of populations; $\beta$ represents the probability of infection rate, which means that each susceptible population randomly infects $\beta$ people every day. The recovery rate $\gamma$ indicates that the infected person recovers or dies with the probability of $\gamma$.

Although the SIR model is simple, the analysis and use of it in many studies generally show that it can capture the trend and overall characteristics of the epidemic. In the traditional SIR model, $\upbeta$ and $\gamma$ are parameters that reflect the characteristics of the epidemic, and they are constants. However, if the parameters are constant, it is often impossible to measure and predict the development trend of epidemics when applied to the real world. Therefore, many studies have regarded them as functions that change over time and used equations to derive them. Considering that during the development of the epidemic, the parameters in the SIR model are changing in real time for different countries and regions. In order to reflect these changes in the parameters of the epidemic model, in this article we propose the TW-SIR prediction model, which can capture, track and predict the dynamic changes of the epidemic parameters in real time. We will introduce this model in detail in the next section.

### Time-window SIR model

**Figure 1:**
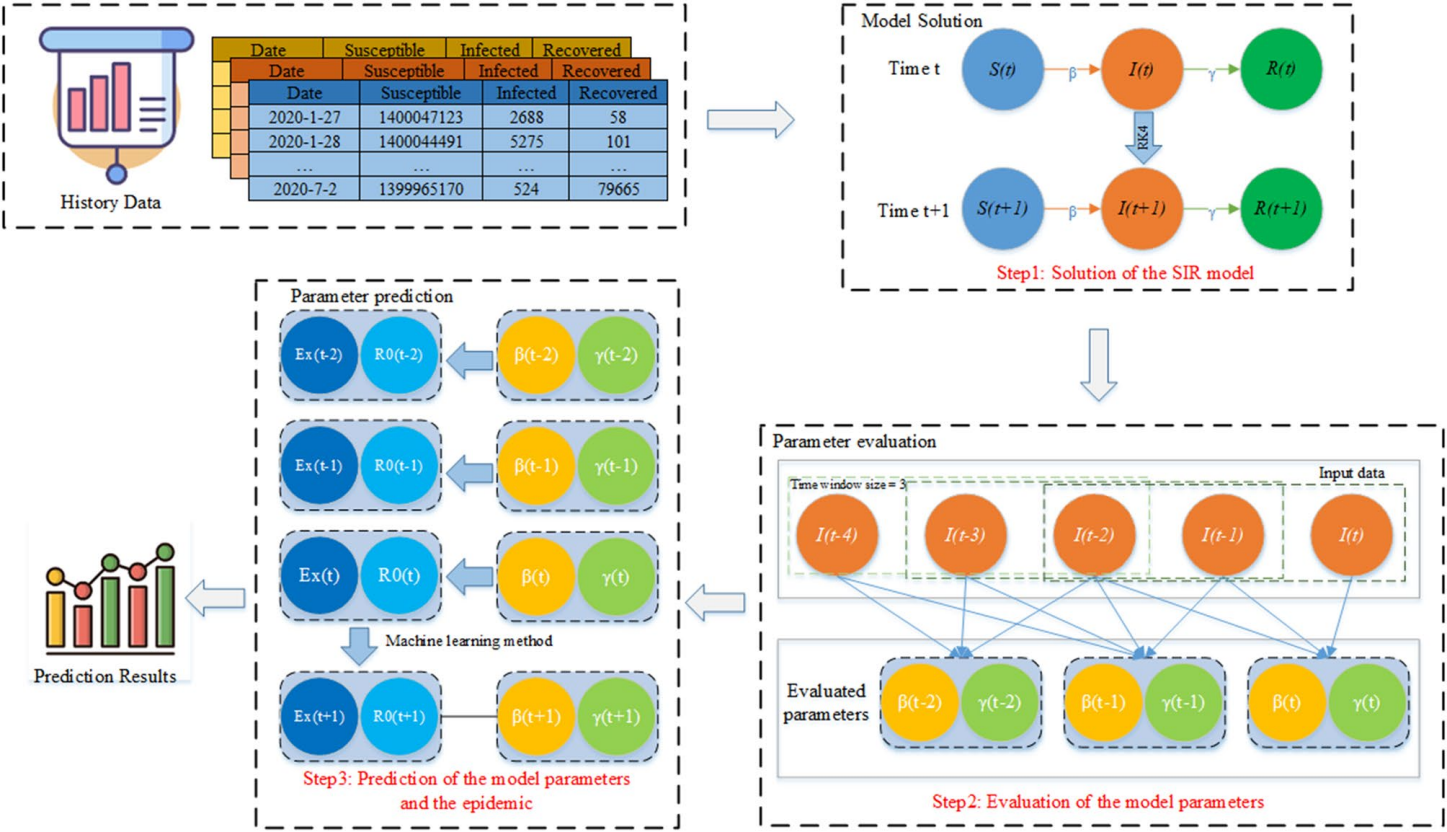
The main workflow of the TW-SIR model.

In order to represent the changes of parameters in the SIR model, we propose a time window-based SIR model (TW-SIR) which splits historical data into a time window segment. The purpose of this method is to capture the real-time changes in ${R}_{0}$ and the exponential growth rate $Ex$. The TW-SIR model is based on the assessment of the changes in the epidemiological parameters of historical data every day through a time window and solves the problem that the formula derivation method cannot be measured in real time. Figure 1 shows the main workflow of the model.

As shown in Fig. 1, the TW-SIR model is mainly composed of three parts: model solution, parameter evaluation and parameter prediction. The detailed process is as follows.

*Step 1: Solution of the SIR model.* First, the historical data input for the TW-SIR model includes the daily number and data of susceptible, infected and recovered populations, and the data are divided according to the time window size. For the data in the specified time window, Runge–Kutta method is used to solve the SIR model numerically.

*Step 2: Evaluation of the model parameters.* Based on the historical data in the time window, the least square method is used to set the initial values of the model parameters, and then the model parameters are traversed and searched to represent the changes of the basic reproduction number ${R}_{0}$ and the exponential growth rate $Ex$ in the historical data.

*Step 3: Prediction of the model parameters and the epidemic.* Based on the existing parameter values obtained from the parameter evaluation, a machine learning method was used to track and predict the future parameter values with the combination of basic reproduction number ${R}_{0}$ and exponential growth rate $Ex$. Finally, the prediction results of the epidemic are returned.

Aim of TW-SIR is to evaluate the changes in the parameters of the epidemic in order to predict the development trend of the epidemic. In the rest of this section, we will describe the contents of each part in detail.

#### Model solution

The function of model solution is to numerically solve SIR model equations to facilitate subsequent parameter evaluation. Because SIR model equations are coupled nonlinear ordinary differential equations, it is difficult to find analytical solutions to the equations. Although it is possible to derive the analytical solution of the equation in implicit form, the solution process is complicated and practical applications have limitations^28^. Compared with analytical solutions, methods such as numerical solutions are more commonly used in such research problems, and these methods are more effective. In this paper, numerical solution method, namely Runge–Kutta method, is used to numerically solve the SIR model. The Runge–Kutta method is a high-precision single-step algorithm, and its classic method is the fourth-order Runge–Kutta method (RK4). RK4 divides the time interval between $t$ and $t+1$ into four subintervals and solves ordinary differential equations by calculating the slope values of these subintervals points and weighting them as the average slope. For the three states of the SIR model, we use RK4 to modify the differential equations in (1–3) into discrete differential equations:

5 $$S\left(t+1\right)=S\left(t\right)+\frac{h({S}_{1}^{^{\prime}}+2{S}_{2}^{^{\prime}}+{2S}_{3}^{^{\prime}}+{S}_{4}^{^{\prime}})}{6}$$

6 $$I\left(t+1\right)=I\left(t\right)+\frac{h({I}_{1}^{^{\prime}}+2{I}_{2}^{^{\prime}}+{2I}_{3}^{^{\prime}}+{I}_{4}^{^{\prime}})}{6}$$

7 $$R\left(t+1\right)=R\left(t\right)+\frac{h({R}_{1}^{^{\prime}}+2{R}_{2}^{^{\prime}}+{2R}_{3}^{^{\prime}}+{R}_{4}^{^{\prime}})}{6}$$

where $\mathrm{h}$ is the step-size, ${S}_{i}^{^{\prime}}$, ${I}_{i}^{^{\prime}}$ and ${R}_{i}^{^{\prime}}$ ($\mathrm{i}=\mathrm{1,2},\mathrm{3,4}$), respectively indicate the slopes of the four subintervals in the interval [t,t + 1] of $S\left(t\right)$, $I\left(t\right)$ and $R\left(t\right)$, which can be calculated by Eqs. (8–11)

8 $$\left\{ {\begin{array}{*{20}l} {S_{1}^{\prime } = - \frac{{\beta S(t)I(t)}}{N}} \hfill \\ {I_{1}^{\prime } = \frac{{\beta S(t)I(t)}}{N} - \gamma I(t)} \hfill \\ {R_{1}^{\prime } = \gamma I(t)} \hfill \\ \end{array} } \right.$$

9 $$\left\{ {\begin{array}{*{20}l} {S_{2}^{\prime } = - \frac{{\beta \left( {S\left( t \right) + \frac{{hS_{1}^{\prime } }}{2}} \right)\left( {I\left( t \right) + \frac{{hI_{1}^{\prime } }}{2}} \right)}}{N}} \hfill \\ {I_{2}^{\prime } = \frac{{\beta \left( {S\left( t \right) + \frac{{hS_{1}^{\prime } }}{2}} \right)\left( {I\left( t \right) + \frac{{hI_{1}^{\prime } }}{2}} \right)}}{N} - \gamma \left( {I\left( t \right) + \frac{{hI_{1}^{\prime } }}{2}} \right)} \hfill \\ {R_{2}^{\prime } = \gamma \left( {I\left( t \right) + \frac{{hI_{1}^{\prime } }}{2}} \right)} \hfill \\ \end{array} } \right.$$

10 $$\left\{\begin{array}{l}{S}_{3}^{^{\prime}}=-\frac{\beta (S\left(t\right)+\frac{h{S}_{2}^{^{\prime}}}{2})(I\left(t\right)+\frac{h{I}_{2}^{^{\prime}}}{2})}{N}\\ {I}_{3}^{^{\prime}}=\frac{\beta (S\left(t\right)+\frac{h{S}_{2}^{^{\prime}}}{2})(I\left(t\right)+\frac{h{I}_{2}^{^{\prime}}}{2})}{N}-\gamma (I\left(t\right)+\frac{h{I}_{2}^{^{\prime}}}{2})\\ {R}_{3}^{^{\prime}}=\gamma (I\left(t\right)+\frac{h{I}_{2}^{^{\prime}}}{2})\end{array}\right.$$

11 $$\left\{\begin{array}{l}{S}_{4}^{^{\prime}}=-\frac{\beta (S\left(t\right)+h{S}_{3}^{^{\prime}})(I\left(t\right)+h{I}_{3}^{^{\prime}})}{N}\\ {I}_{4}^{^{\prime}}=\frac{\beta (S\left(t\right)+h{S}_{3}^{^{\prime}})(I\left(t\right)+h{I}_{3}^{^{\prime}})}{N}-\gamma (I\left(t\right)+h{I}_{3}^{^{\prime}})\\ {R}_{4}^{^{\prime}}=\gamma (I\left(t\right)+h{I}_{3}^{^{\prime}})\end{array}\right.$$

Through the above equations, $\beta$ and $\gamma$ are substitute into the SIR model to solve the model numerically. The three functions of $S\left(t\right)$, $I\left(t\right)$ and $R\left(t\right)$ satisfy Eq. (4).

#### Parameter evaluation

The parameters evaluation part is mainly to characterize the change of the infected rate $\beta$ and the recovered rate $\gamma$ over time in the historical data, so as to facilitate subsequent parameter prediction. Firstly, the historical data is divided according to the size of the time window, then an initial values of the model parameters are set within the time window, and then traverse the search for the model parameters, and finally get the best model parameters for each day through evaluation. A time-dependent $\beta (t)$ and $\gamma (t)$ functions are used to instead of $\beta$ and $\gamma$ in the SIR model, which can be obtained

12 $$\frac{dS(t)}{dt}=-\frac{\beta (t)I(t)S(t)}{N}$$

13 $$\frac{dI(t)}{dt}=\frac{\beta (t)I(t)S(t)}{N}-\gamma (t)I(t)$$

14 $$\frac{dR(t)}{dt}=\gamma (t)I(t)$$

where $\beta (t)$ and $\gamma (t)$ are functions with time $t$ as an independent variable rather than constants. Due to the government action on infection prevention and control for COVID-19 and awareness of the population on COVID-19, $\beta (t)$ and $\gamma (t)$ change in real time. In order to measure this change, the time series data set is divided into time windows of size W, and then use the optimal parameter solution in the time window as the evaluation value. For historical data at time $t$, its time window is $\{{w}_{t} ,0\le \mathrm{t}\le \mathrm{T}-1\}$, we can get the following equation:

15 $$\beta \left(t\right)=opt\left\{{\beta }_{{w}_{t}}\right\},{w}_{t}=\left[t-w+1,t\right]$$

16 $$\upgamma \left(t\right)=opt\left\{{\upgamma }_{{w}_{t}}\right\},{w}_{t}=\left[t-w+1,t\right]$$

Among them, ${\beta }_{{w}_{t}}$ and ${\upgamma }_{{w}_{t}}$ represent a certain parameter solution in the SIR model at time $t$ in the historical data with a time window size of $w$. In order to obtain the optimal solution $opt\left\{{\beta }_{{w}_{t}}\right\}$ under the time window $w$, two steps are applied in calculating it through search: firstly, determine the initial values of the model parameters and secondly perform traversal search on the model parameters. The first is the determination of the initial values of the model parameters. In the early stages of the epidemic, the proportion of the number of infected and cured population in the population is negligible. We can regard the susceptible number $S\left(t\right)$ and the total population $N$ as approximately equal, so the differential Eq. (2) can be written as the Eq. (17):

17 $$\frac{dI(t)}{dt}=\left(\beta -\gamma \right)I(t)$$

Then we can get the analytical solution of the model through the above equation, as shown in Eq. (18):

18 $$I\left(t\right)={e}^{\left(\beta -\gamma \right)t}$$

where, the number of infected people is an exponential function that changes over time, and then the least squares method is used to retrospectively fit the actual data of the epidemic to obtain the initial values ${\beta }_{0}$ and ${\gamma }_{0}$ of the parameter. The initial value obtained can evaluate the characteristics of the early stage of the epidemic, but a simple exponential growth model cannot fully reflect the full picture of the epidemic and a more accurate estimation needed. Therefore, based on the initial values, total number of confirmed COVID-19 cases and model numerical solution methods are used to traverse the model parameters.

Given the data within a specified time window $\left\{C\left(t\right),R\left(t\right),D\left(t\right), 0\le t\le T-1\right\}$ ($C\left(t\right),R\left(t\right)$ and $D\left(t\right)$ are respectively the cumulative number of COVID-19 cases, cumulative number of cured COVID-19 cases, and cumulative number of death cases per day), Eq. (19) is used to calculate the actual daily number of infections $I(t)$:

19 $$I\left(t\right)=C\left(t\right)-R\left(t\right)-D(t)$$

After getting the daily actual number of infected people, we use the RK4 method to find the numerical solution of the model, which is the predicted number of infected people $I\left(t\right)$. In order to evaluate the parameters $\beta$ and $\gamma$, the following equation is used to calculate the MSE (mean squared error) of the predicted result:

20 $$MSE\left(\beta ,\gamma \right)=\frac{1}{T}\sum_{t}^{T}{(\widehat{I}\left(t\right)-I\left(t\right))}^{2}$$

In order to get the optimal size of time window, the size of time window is set from 3 to 30 to be tested and the accumulated forecast error is used to evaluate the accuracy and effectiveness of the forecast under each time window. ${Error}_{w}$ is the accumulated prediction error under the time window $w$, and the equation is shown as following:

21 $${Error}_{w}=\frac{1}{T-W}\sum_{t=w-1}^{T}\frac{\left|\widehat{I}\left(t\right)-I\left(t\right)\right|}{I\left(t\right)}$$

In the process of searching for model parameters, it takes too much time if a grid search is applied and it is easy to fall into the local optimum. To overcome this problem, in this article an optimized search method is used. First, we assume that the value of $\beta$ is greater than the value of $\gamma$ in the early stage of the epidemic, because this is necessary to ensure that the epidemic infection continues^29^, namely the value of ${R}_{0}$ is greater than 1 and estimate the initial parameter values ${\beta }_{0}$ and ${\gamma }_{0}$ using Eqs. (17) and (18). Based on the initial values ${\beta }_{0}$ and ${\gamma }_{0}$, we set the size of search step and the size of search interval. Then RK4 is used to solve the model by using Eq. (6). Finally, the MSE for ${\beta }_{{w}_{t}}$ and ${\gamma }_{{w}_{t}}$ are calculated and the ${\beta }_{{w}_{t}}$ and ${\gamma }_{{w}_{t}}$ with minimize of MSE are as Optimal parameters. The detailed steps of our parameter evaluation based on time window are shown in Algorithm 1.
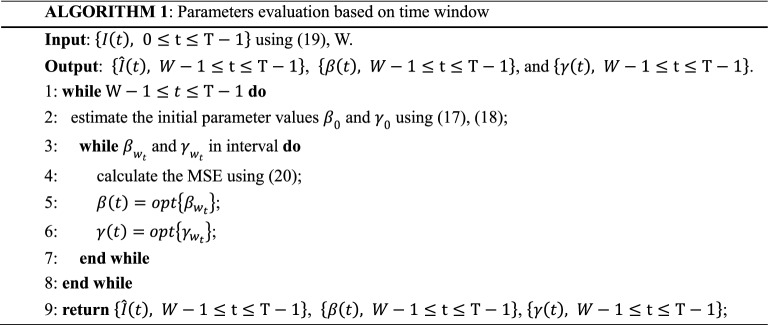


After getting $\beta \left(t\right),\gamma \left(t\right)\{\beta \left(t\right),\gamma \left(t\right), w-1\le t\le T-1\}$, machine learning methods can be applied to predict the time change of the infection coefficient and the cure coefficient and predict the future development trend of the epidemic.

#### Parameter prediction

Parameters prediction is to predict the subsequent model parameters based on the changes over time of the model parameters obtained from the previous part of the parameter evaluation. In this section, the polynomial regression algorithm widely used in machine learning is applied to track and predict $\beta \left(t\right)$ and $\gamma \left(t\right)$. It is difficult to accurately directly predict $\beta \left(t\right)$ and $\gamma \left(t\right)$ because of value fluctuations. Therefore, this paper proposes a new prediction method, using the method of predicting the ${R}_{0}$ and exponential growth rate $\mathrm{Ex}\left(t\right)$ to calculate them, which their changing curve is easier to predict in the development of the epidemic. The Basic reproduction number ${R}_{0} \mathrm{also}$ reflects the development of the epidemic. It can also be regarded as a function over time ${R}_{0}\left(t\right)$ which can be obtained by using Eq. (22):

22 $${R}_{0}\left(t\right)=\frac{\beta \left(t\right)}{\gamma \left(t\right)}$$

In order to get $\beta \left(t\right)$ and $\gamma \left(t\right)$, we define an exponential growth rate index $\mathrm{Ex}\left(t\right)$ according to the exponential growth model of Eq. (18), which is shown in the following equation:

23 $$Ex\left(t\right)=\beta \left(t\right)-\gamma \left(t\right)$$

where the predicted basic reproduction number is $\widehat{{R}_{0}}(t)$, and the predicted exponential growth rate is $\widehat{\mathrm{Ex}}(t)$. Through polynomial regression, they can be written in the following form:

24 $$\begin{aligned} \widehat{{R_{0} }}\left( t \right) & = a_{0} + a_{1} R_{0} \left( {{\text{t}} - 1} \right) + a_{2} \left( {R_{0} \left( {{\text{t}} - 1} \right)} \right)^{2} + \cdots + ~a_{n} \left( {R_{0} \left( {{\text{t}} - 1} \right)} \right)^{n} \\ & = \mathop \sum \limits_{{i = 0}}^{n} a_{i} \left( {R_{0} \left( {{\text{t}} - 1} \right)} \right)^{i} \\ \end{aligned}$$

25 $$\begin{aligned} \widehat{{{\text{Ex}}}}\left( t \right) & = b_{0} + b_{1} {\text{Ex}}\left( {{\text{t}} - 1} \right) + b_{2} \left( {{\text{Ex}}\left( {{\text{t}} - 1} \right)} \right)^{2} + \cdots + {\text{~}}b_{m} \left( {{\text{Ex}}\left( {{\text{t}} - 1} \right)} \right)^{m} \\ & = \mathop \sum \limits_{{j = 0}}^{m} a_{j} \left( {{\text{Ex}}\left( {{\text{t}} - 1} \right)} \right)^{j} \\ \end{aligned}$$

n and m are the order of $\widehat{{R}_{0}}\left(t\right)$ and $\widehat{\mathrm{Ex}}\left(t\right)$ polynomials (n, m ≥ 2), ${a}_{i} (i=\mathrm{0,1},\dots ,n)$ and ${b}_{j} (j=\mathrm{0,1},\dots ,m)$ are the coefficients of these two polynomial functions. In order to determine the coefficient and order of the polynomial function, the most widely used least squares method (OLS) to evaluate the prediction results. At the same time, in order to ensure that the model is under-fitting and reflect the real-time changes of the epidemic, Time window method mentioned in the previous section is used to solve the following optimization problems:

26 $$\mathrm{min}\sum_{t=T-W}^{T-1}{(\widehat{{R}_{0}}\left(t\right)-{R}_{0}\left(\mathrm{t}\right))}^{2}$$

27 $$\mathrm{min}\sum_{t=T-W}^{T-1}{(\widehat{\mathrm{Ex}}\left(t\right)-\mathrm{Ex}\left(\mathrm{t}\right))}^{2}$$

W is the size of the time window. The coefficients and orders of the polynomial can be obtained by solving the objective optimization function, such as ${a}_{i},i=\mathrm{0,1},\dots ,n$, and ${b}_{j},j=\mathrm{0,1},\dots ,m$. After obtained these coefficients, $\widehat{{R}_{0}}(t)$ and $\widehat{\mathrm{Ex}}(t)$ at time $t=T$ can be obtained through the Eqs. (24, 25), and then the predicted infection rate $\upbeta \left(\mathrm{t}\right)$ and the predicted recovery rate $\gamma \left(t\right)$ can be calculated by using Eqs. (28) and (29), namely:

28 $$\widehat{\beta }\left(t\right)=\frac{\widehat{\mathrm{Ex}}(t)\widehat{{R}_{0}}(t)}{1-\widehat{{R}_{0}}(t)}$$

29 $$\widehat{\gamma }\left(t\right)=\frac{\widehat{\mathrm{Ex}}(t)}{1-\widehat{{R}_{0}}(t)}$$

Now we have got $\widehat{\beta }(t)$ and $\widehat{\gamma }(t)$, and then through the model solution method in “Model solution”, the number of infections $\left\{\widehat{I}\left(t\right), t>T\right\}$ in the subsequent epidemic can be predicted.

## Numerical results

### Data sources

In this paper, we gathered epidemiological data from Johns Hopkins University^30^. Project data is available on the open source GitHub site, and the life cycle of the project is continuous^31^. The data include the various countries from January 23, 2020 up to now. The daily cumulative number of confirmed cases, cumulative death cases, and cumulative cured cases in the region. Taking China as an example, Table 1 shows the details of the data we used. In this article, we use the data of seven countries including China, South Korea, France, Spain, Italy, Germany and Brazil as our data set. In addition, in order to verify that our method is applicable to different epidemics, we also gathered the SARS epidemic data of Beijing, China from April 20, 2003 to June 23, 2003 from the website of the Ministry of Health of China, and the format of the data is the same as in Table 1. Table 2 shows the COVID-19 data for China, South Korea, France, Spain, Italy, Germany, and Brazil, and the time frame of the 2003 Beijing SARS data.

**Table 1:** COVID-19 data in China.

Date	Confirmed	Deaths	Recovered
2020/01/27	2877	131	58
2020/01/28	5509	133	101
…	…	…	…
2020/07/01	84,816	4641	79,650
2020/07/02	84,830	4641	79,665

**Table 2:** Data set description.

Country or Province	Date	Type of epidemic
China	2020/01/27–2020/07/02	COVID-19
Korea South	2020/02/20–2020/07/02	COVID-19
Italy	2020/02/26–2020/07/02	COVID-19
Spain	2020/02/26–2020/07/02	COVID-19
Brazil	2020/02/26–2020/07/02	COVID-19
Germany	2020/02/27–2020/07/02	COVID-19
France	2020/02/28–2020/07/02	COVID-19
Beijing province in China	2003/04/20–2003/06/23	SARS

### Parameter setup

**Figure 2:**
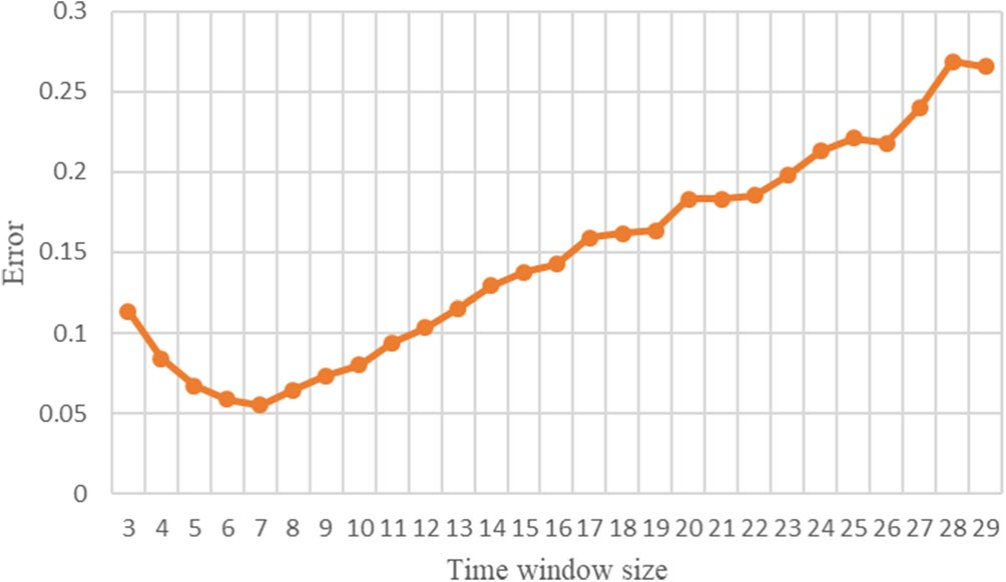
Changes in prediction error when the time window size is 3–29.

1. Determination of window value $W$
  Different time window sizes are used in the experiment, which scope is from 3 to 30. Figure 2 shows the cumulative forecast error of China under different time windows calculated according to Eq. (21). It can be found that there is a time window that minimizes the cumulative forecast error, that is, $W = 7$.
  For every country in the data set, the respective optimal time window size is shown in Table 3.
2. Parameter evaluation
  After determining the appropriate time window size, Algorithm 1 is used to evaluate the model parameters. When using polynomial regression to predict the parameters $\beta \left(t\right)$ and $\gamma \left(t\right)$, we set initial order of the polynomial to 2, that is, $n=m=2$. Because $\beta \left(t\right)$ and $\gamma \left(t\right)$ are non-negative, if their value is less than 0 in the regression calculation, we set them to 0. The stopping condition in the model solving process is $I(t)\le 0$. Finally, we use model solving methods to predict the development trend of the epidemic.

**Table 3:** The optimal time window size of each country or region in the data set.

Country	China	Korea South	Italy	Spain	Brazil	Germany	France	Beijing
Optimal time window size	7	7	4	4	6	6	5	7

### Experiment and result analysis

In order to illustrate the scientificity and effectiveness of the TW-SIR model, we will present and analyze the three research questions (RQ1, RQ2 and RQ3) in this section.

#### RQ1 experiment results

In the epidemic model, a very important question is when the epidemic will end. To answer this question, one commonly used indicator is the basic reproduction number ${R}_{0}$, which is defined as the average of how many other people an infected person will transmit the disease to before they recover. In the TW-SIR prediction model, ${R}_{0}\left(t\right)$ is a time-dependent function. If ${R}_{0}\left(t\right)>1$, the epidemic will spread quickly and infect a certain percentage of the total population N. On the contrary, if ${R}_{0}\left(t\right)<1$, the epidemic will eventually be brought under control and end. Therefore, by observing the changes in ${R}_{0}\left(t\right)$ and predicting the future $\widehat{{R}_{0}}\left(t\right)$, the development trend of the epidemic and whether the control measures of the epidemic are effective can be known. At the same time, in this paper, an indicator exponential growth rate $Ex\left(t\right)$ is used, that is, the difference between $\beta \left(t\right)$ and $\gamma \left(t\right)$, to measure the exponential growth trend of the epidemic, which also reflects the changing trend of the epidemic. When $\mathrm{Ex}(\mathrm{t})>0$, it means that the infection speed of the epidemic is faster than the cure. On the contrary, the number of people infected by the epidemic is gradually cured and the epidemic is gradually coming to an end. Firstly, we applied TW-SIR model to the historical data of COVID-19 in China, South Korea, Italy, Spain, Brazil, Germany and France from January 27 to July 2, 2020 to measure ${R}_{0}\left(t\right)$ and $Ex\left(t\right)$. We compare TW-SIR prediction model with the measurement method based on formula derivation proposed in^22^. Tables 4 and 5, respectively summarize the basic reproduction number ${R}_{0}$ and exponential growth rate $Ex$ measured using the TW-SIR model and the formula derivation method used in literature^22^. It can be seen from the table that the parameter values measured based on the TW-SIR model are closer to the actual situation, while the formula derivation method has outliers inconsistent with the actual situation, such as too large or too small.

**Table 4:** The measurement of the reproduction number and the exponential growth rate according to TW-SIR model.

	China	Korea South	Italy	Spain	Brazil	Germany	France
${R}_{0}$ average value	2.285	2.428	3.407	4.217	5.136	4.029	4.091
${R}_{0}$ min value	0.031	0.143	0.125	0.167	0.125	0.013	0.043
${R}_{0}$ max value	49	61	41	50	44	67	42
$Ex$ average value	− 0.0138	0.0412	0.0512	0.075	0.1045	0.0518	0.0681
$Ex$ min value	− 0.31	− 0.08	− 0.07	− 0.09	− 0.11	− 0.78	− 0.22
$Ex$ max value	0.48	0.77	0.98	0.62	0.5	0.77	0.98

**Table 5:** The measurement of the reproduction number and the exponential growth rate according to the formula derivation method.

	China	Korea South	Italy	Spain	Brazil	Germany	France
${R}_{0}$ average value	4.551	5.094	5.469	14.278	7.627	11.599	− 1.885
${R}_{0}$ min value	− 0.25	− 15.71	− 70	− 3.35	0.16	0	− 719
${R}_{0}$ max value	134.4	195	229	287.7	156.57	372.125	257.82
$Ex$ average value	0.0016	0.0655	0.0968	0.0896	0.1325	0.0981	0.0995
$Ex$ min value	− 0.3468	− 0.1699	− 0.0704	− 1	− 0.3929	− 0.7333	− 0.7143
$Ex$ max value	0.9280	3.5789	5.33	1.75	2.25	3	4

**Figure 3:**
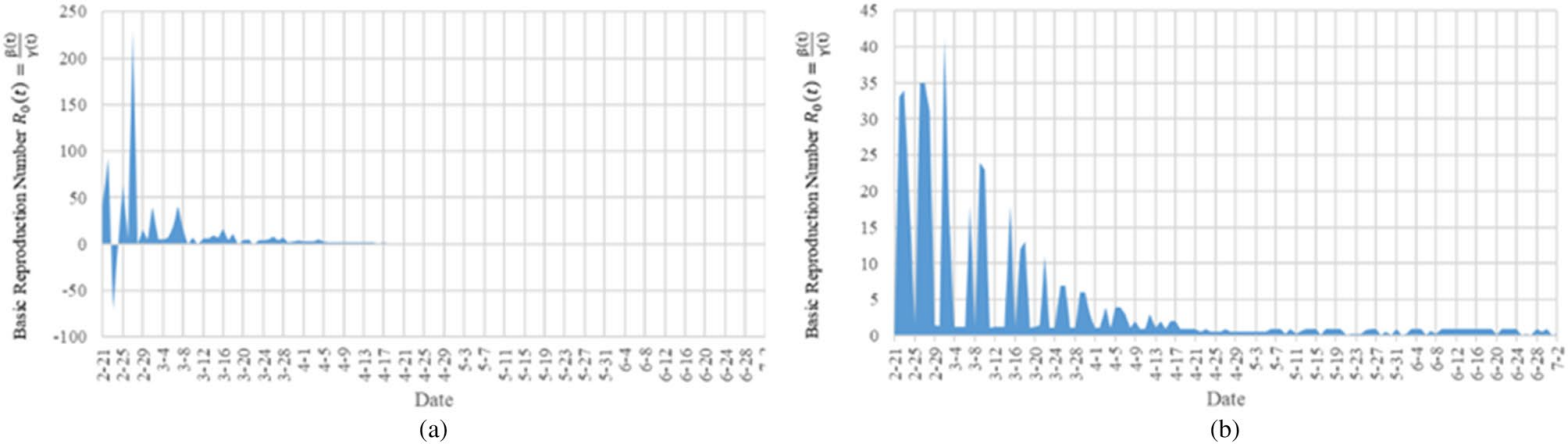
Basic reproduction number ${R}_{0}\left(\mathrm{t}\right)$ in Italy.

Figure 3a shows the result of using the data to measure ${R}_{0}\left(t\right)$ method in the literature^22^, and Fig. 3b is the result of using the TW-SIR model to measure ${R}_{0}\left(\mathrm{t}\right)$. All date starts from February 21, 2020 in the two figures. ${R}_{0}\left(\mathrm{t}\right)$ in Fig. 3a has reached two hundred, and there are negative values, which is obviously not true. We can also see from Fig. 3b that the value of the ${R}_{0}\left(\mathrm{t}\right)$ is much smaller and more in line with the actual situation. In addition, in Fig. 3b, it can be seen that there is a turning point of ${R}_{0}\left(\mathrm{t}\right)<1$ on April 19, 2020, that is, the epidemic situation in Italy reaches its peak at this moment. After April 19, 2020, ${R}_{0}\left(\mathrm{t}\right)$ remains at a level less than 1, which means that the number of infected people $I(t)$ will decrease and will lead to the end of the Italian epidemic. TW-SIR model can accurately measure the time when ${R}_{0}\left(\mathrm{t}\right)<1$ and the measured value is close to the actual situation. At the same time, our results are similar to those measured in most literatures^32^, which shows the effectiveness of TW-SIR model to measure ${R}_{0}\left(\mathrm{t}\right)$.

**Figure 4:**
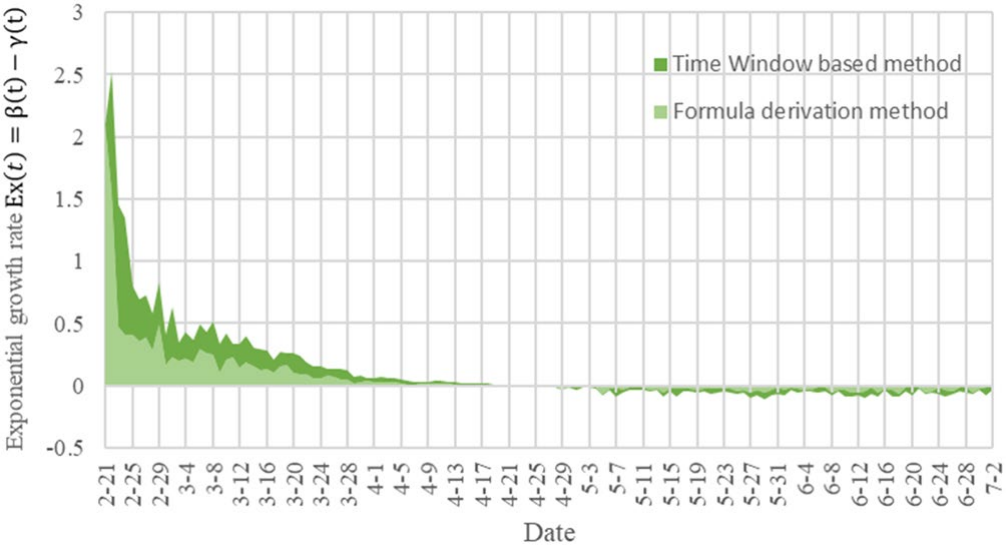
The result of the exponential growth rate $Ex(t)$ in Italy from February 21 to July 2, 2020. (The dark green curve represents the measurement result of our proposed TW-SIR prediction model, and the light green curve is the formula-based method used in^22^).

Similarly, Fig. 4 shows the results of TW-SIR model and formula derivation method in measuring the exponential growth rate $Ex(t)$. The exponential growth rate $Ex(t)$ calculated by the two methods can reflect the development and changes of the epidemic, and the overall trend is roughly the same, and both can measure the peak time of the epidemic. However, the $Ex(t)$ value calculated based TW-SIR model includes the value calculated based on the formula derivation method, which can more clearly reflect the change of the exponential growth rate.

#### RQ2 experiment results

**Figure 5:**
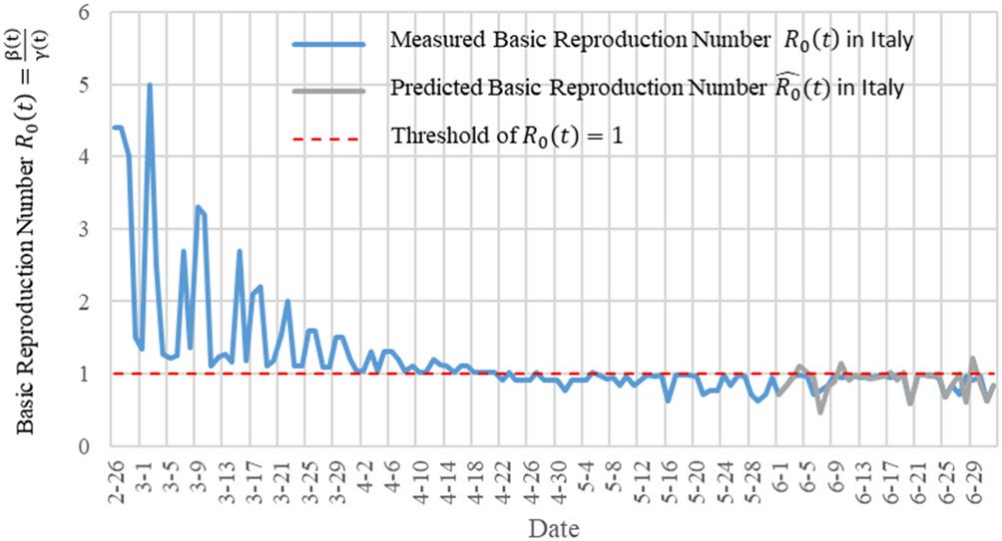
${R}_{0}\left(\mathrm{t}\right)$ and the predicted $\widehat{{R}_{0}}\left(t\right)$ in Italy measured by the TW-SIR prediction model.

Figure 5 shows the measured ${R}_{0}\left(\mathrm{t}\right)$ and the predicted $\widehat{{R}_{0}}\left(t\right)$ in Italy by using TW-SIR model. The blue curve is the measured ${R}_{0}\left(\mathrm{t}\right)$, from February 26, 2020 to July 2, 2020. The gray curve is the predicted $\widehat{{R}_{0}}\left(t\right)$ from June 1, 2020 to July 2, 2020. The red dotted line is the threshold value representing the $\widehat{{R}_{0}}\left(t\right)=1$. We can see that ${R}_{0}$ in Italy was almost the same as ${R}_{0}$ in China in the early stages of the epidemic. From the figure that ${R}_{0}$ is a turning point around April 19, which means a peak of the epidemic. Compared with China, Italy has a relatively long time to enter the peak, which may be caused by different prevention and control strategies.

**Figure 6:**
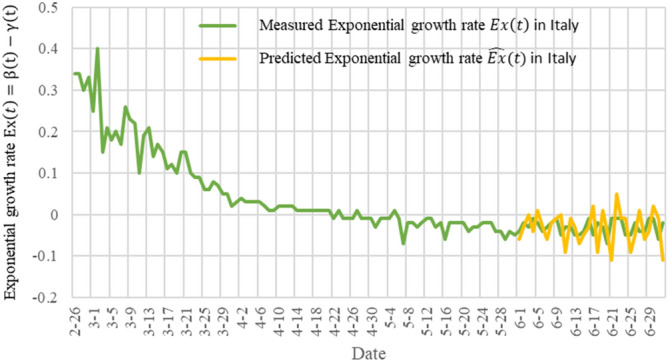
The basic number of infections $Ex(t)$ and the predicted basic number of infections $\widehat{Ex}(t)$ of COVID in Italy measured by the TW-SIR prediction model.

In Fig. 6, we show the exponential growth rate $Ex(t)$ measured by Italy and the predicted exponential growth rate $\widehat{\mathrm{Ex}}(t)$. The green curve is the measured exponential growth rate $Ex(t)$, from February 26, 2020 to July 2, 2020. The yellow curve is the predicted exponential growth rate $\widehat{Ex}(t)$, from June 1, 2020 to July 2, 2020. In Fig. 6, the exponential growth rate of the Italian epidemic has approached zero. If this situation remains, the number of infected persons will decrease and the epidemic will be faded. But due to the changes of temperatures, government's epidemic control measures and people's awareness, there is a second wave of infection from August, 2020, which we'll discuss later. Figures 5 and 6 show that TW-SIR model accurately predicted the changes of $\widehat{{R}_{0}}\left(t\right)$ and $\widehat{\mathrm{Ex}}(t)$ from June 1 onwards, which shows that our parameter prediction method is effective.

**Figure 7:**
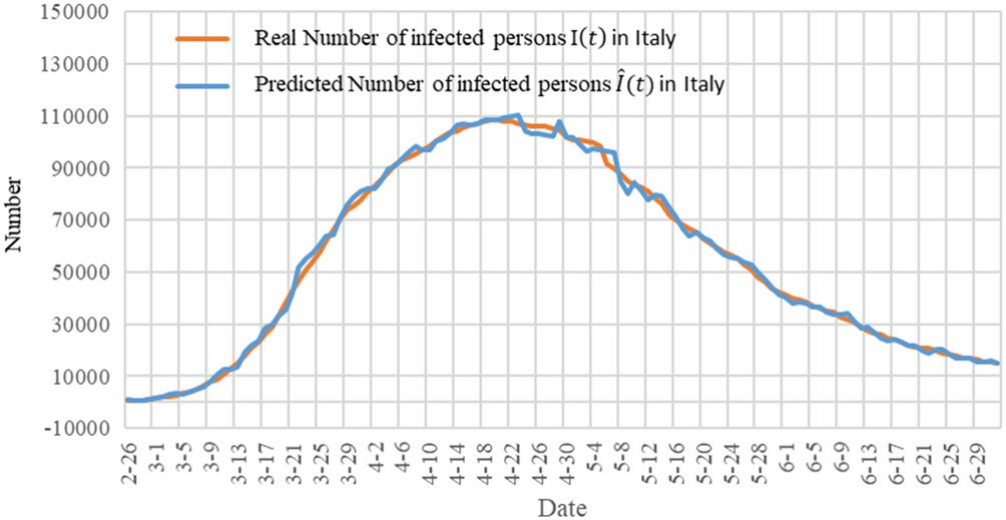
A single-day forecast of the number of infections in Italy. The orange curve represents the actual number of infections $I(t)$ in Italy, and the blue curve represents the predicted number of infections $\widehat{I}(t)$.

In order to show the accuracy of our model, we show the prediction results of our model for the next day (single-day forecast) in Fig. 7. The orange curve in the figure represents the actual number of infections $I(t)$ in Italy, and the blue curve represents the predicted number of infections $\widehat{I}(t)$. The figure shows that the predicted curve is very close to the actual data curve.

**Figure 8:**
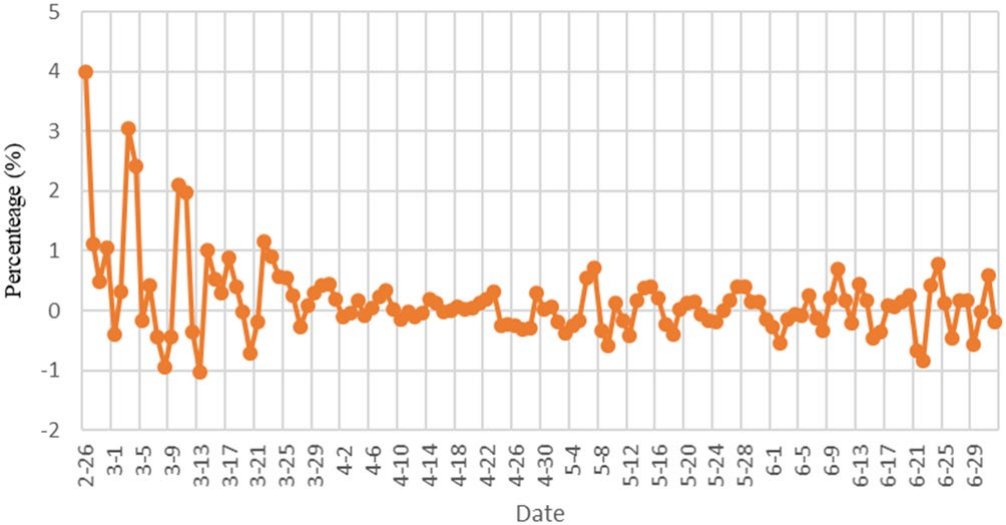
The forecast error of the single-day forecast of the number of infections in Italy.

We further tested the accuracy of our prediction and calculated the error of the single-day prediction of the number of infected people, as shown in Fig. 8. The error rate of the predicted number of infected people is all within 5%, which shows that our model can accurately predict the number of infected people next day.

Judging from the results of applying TW-SIR model to the data of epidemic in China and Italy, the model can effectively measure the real-time changes of parameters during the development of the epidemic, including the basic reproduction number of the epidemic and the exponential growth rate of the development of the epidemic, as well as the development trend of the epidemic follow up and forecast.

#### RQ3 experiment results

From September 2020 into the autumn and winter season, many countries have a second wave of COVID-19 infections. We applied the TW-SIR model to the data from August to October 2020 for seven countries in the data set, and the measurement results are shown in the Table 6.

**Table 6:** The measurement of the reproduction number and the exponential growth rate according to TW-SIR model.

	China	Korea South	Italy	Spain	Brazil	Germany	France
${R}_{0}$ average value	1.009	0.581	1.551	1.272	0.97	1.576	1.293
$Ex$ average value	− 0.0074	− 0.03226	0.0235	0.033	0.0058	0.0187	0.0342
$I(t)$ average value	374	2524	45,917	496,275	428,226	23,907	386,586

In South Korea and Brazil, the ${R}_{0}$ value was less than 1, indicating a downward trend in the number of infections from September to October. For the exponential growth rate, only China and South Korea's exponential growth rate is less than 0, and the average number of infected persons $I(t)$ in these two countries is very small, which means that the development trend of the epidemic in these two countries is in a relatively stable state for a long time. Italy, Spain, Germany and Brazil have all seen a second wave of attacks, and the number of cases is rising.

**Figure 9:**
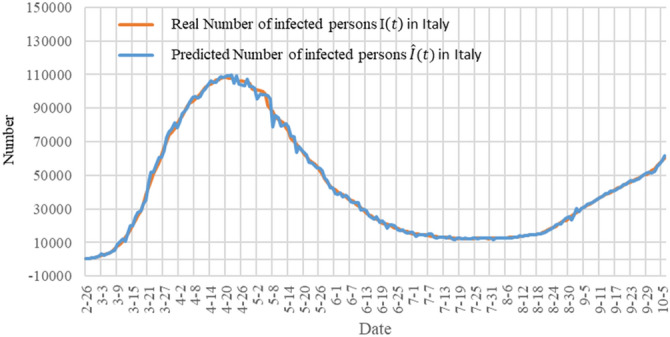
A single-day forecast of the number of infections in Italy from February to October 2020.

In Italy, for example, Fig. 9 shows the trend change in the number of existing infections after the TW-SIR model was applied. The orange line is the actual number of infections, and the blue line is the predicted change in the number of infections. As can be seen from the figure, the number of existing infections showed a slow decline from July to early August 2020. The average value of ${R}_{0}$ obtained by using the TW-SIR model during this period was 0.333, while the number of infected people gradually increased from August, with the average value of ${R}_{0}$ being 1.551. The TW-SIR model successfully predicted the trend of the second wave of infection.

**Figure 10:**
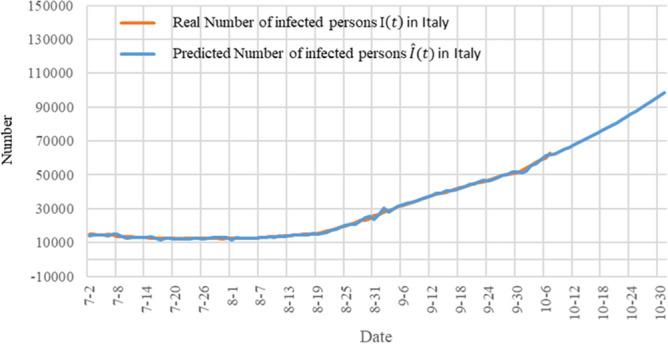
A forecast of the number of infections in Italy from October 2020.

The trend of the number of infections in Italy from July to October shows an increase in the number of infections caused by the second wave. The blue line at the back end is the TW-SIR prediction curve (Fig. 10).

## Discussion

Since the outbreak began in China, COVID-19 has spread to many countries and regions around the world. There have been 37,213,592 confirmed cases in 188 countries and regions on 11 October 2020. Different countries and regions have taken different measures to prevent and control the epidemic, such as closing cities, closing schools and quarantining people at home. As a result, the epidemic has developed at different levels. In previous studies, constants are usually used to measure parameters in epidemic transmission models^1,33–35^, but it is difficult to measure the dynamic and real-time evolution of the epidemic. Different from the fixed parameters of the traditional SIR model, we use the time window to measure the model parameters dynamically and propose the TW-SIR model based on the time window. The advantage of the TW-SIR model is that it is more in line with the actual dynamic measurement of epidemic parameters. We applied the proposed TW-SIR model to historical data from 27 January to 2 July 2020 for China, South Korea, Italy, Spain, Brazil, Germany and France to measure the basic number of infections ${R}_{0}(t)$ and the exponential growth rate $Ex(t)$. Compared with the formula measurement method proposed in literature^22^, the measurement results of the TW-SIR model are closer to the reality.

As for the ${R}_{0}$ values assessed, as shown in Table 6, Brazil has the highest ${R}_{0}$ mean (5.136) and China has the lowest ${R}_{0}$ mean (2.285) among the seven countries. Liu Ying et al. reviewed the ${R}_{0}$ of COVID-19 in 12 studies and found that the estimated average ${R}_{0}$ of COVID-19 was about 3.28, with a median of 2.79 and an IQR of 1.16^32^. Our ${R}_{0}$ of seven countries average measurement results for the average, the ${R}_{0}$ value of 3.656, and studies have found similar worth pointing out that measured value ${R}_{0}$ is large, it is because we only select the parameter measurement, seven countries outbreak spread of COVID—19th in various countries have differences, further studies are needed to confirm this measurement index of growth also illustrates some problems, in the selection of seven countries, only China's exponential growth rate is negative, for other countries outbreak development degree and not to a fairly low level. This also shows that China has done a good job in prevention and control measures.

**Figure 11:**
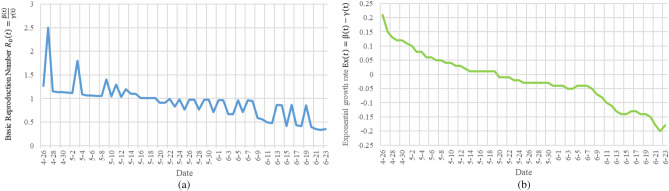
${R}_{0}\left(\mathrm{t}\right)$ and $\mathrm{Ex}\left(\mathrm{t}\right)$ of the SARS in Beijing, China in 2003.

In order to show that our method is applicable to different epidemics, we also used epidemiological data for SARS in Beijing, China, from April 20, 2003 to June 23, 2003. Figure 11 shows the change curves of ${R}_{0}$ and exponential growth rate $Ex$ of the SARS in Beijing, China in 2003. Among them, the average number of basic infections transmitted by SARS in Beijing was 2.099 and the average exponential growth rate was − 0.02046. Compared with the spread of COVID-19 in China, ${R}_{0}$ of SARS in the early stage of infection is about half of ${R}_{0}$ of COVID-19, and the exponential growth rate is about a quarter of that of COVID-19. This is consistent with the actual situation^32^, indicating that COVID-19 spread more violently than the SARS in 2003. At the same time, the average rate of exponential growth was negative during the whole epidemic period, which ensured the end of SARS epidemic.

Our model has several limitations when it comes to parameter measurement and trend prediction of epidemic transmission processes. First, the model did not take account of asymptomatic infected persons because they are difficult to obtain and may be inaccurate. Second, another limitation of our study is that the methods we use in each part of the model may not be optimal, and there are better methods for solving the model and predicting the parameters.

## Conclusion

With the outbreak of the epidemic in other countries and regions, COVID-19 has swept the world. In this study, we proposed a TW-SIR prediction model which is able to reflect the real-time trend of the epidemic in the process of infection for different areas, different policies and different epidemic diseases. Machine learning methods are applied to predict the basic number of infections ${R}_{0}$ and the exponential growth rate of the epidemic $Ex$. And we conducted mathematical and numerical analyses for COVID-19. The numerical results shows that the model can effectively measure the real-time changes of parameters during the spread of epidemics, including the basic number of infections ${R}_{0}\left(\mathrm{t}\right)$ and exponential growth rate $Ex(t)$. And error rate of predicting the number of COVID-19 infections in a single day is within 5%. In general, the measurement of these parameters is of great significance for understanding the spread of COVID-19 and guiding the designation of control strategies and measures.

In addition, many countries have a second wave of COVID-19 infections from September 2020 into the autumn and winter season. From our analysis of outbreak data in Italy from July to October 2020, we found that the TW-SIR model can be adapted to the second peak of COVID-19. In terms of the parameters we measure, China and South Korea have maintained low ${R}_{0}$ and exponential growth rates, while Italy, Spain, Brazil, Germany and France are mostly still on the rise. This means that the epidemic prevention and control measures need to be more stringent to ensure that the epidemic does not get out of control.

Last but not least, the TW-SIR model can also be applied in different epidemics such as SARS based on the experimental results. Although we lack the knowledge on the data of asymptomatic infection cases, our research results will provide some advice for the follow-up epidemic prevention and control.

## Data Availability

Publicly available datasets can be found here: https://github.com/CSSEGISandData/COVID-19. SARS data in Beijing is Available at: https://web.archive.org/web/20030801083745/http://www.moh.gov.cn/zhgl/yqfb/index.htm. And our code and experimental data is publicly available at: https://github.com/Rambo55555/TW-SIR.
